# Combining *I148M* and *E167K* variants to improve risk prediction for nonalcoholic fatty liver disease in Qingdao Han population, China

**DOI:** 10.1186/s12944-019-0992-9

**Published:** 2019-02-09

**Authors:** Li-Zhen Chen, Hong-Yun Ding, Shou-Sheng Liu, Qun Liu, Xiang-Jun Jiang, Yong-Ning Xin, Shi-Ying Xuan

**Affiliations:** 1 College of Medicine and Pharmaceutics, Ocean University of China, Qingdao, 266003 China; 2 Department of Gastroenterology, Qingdao Municipal Hospital Group, Qingdao, 266011 China; 3 Central Laboratories, Qingdao Municipal Hospital Group, Qingdao, 266011 China; 4 Medical College, Qingdao University, Qingdao, 266021 China

**Keywords:** Nonalcoholic fatty liver disease, PNPLA3, TM6SF2, Additive effect

## Abstract

**Background:**

*PNPLA3 I148M* variant and *TM6SF2 E167K* variant are recognized as the major genetic modifiers of nonalcoholic fatty liver disease (NAFLD). The present study sought to evaluate the potential additive effect of the two variants on the risk of NAFLD in Qingdao Han Population, China.

**Methods:**

We genotyped *PNPLA3 I148M* variant and *TM6SF2 E167K* variant in a cohort of 512 unrelated NAFLD patients and 451 healthy controls by sequencing and polymerase chain reaction analysis. In addition, serum lipid profiles and liver enzymes were determined by standard clinical laboratory methods.

**Results:**

The minor allele frequencies were 45.48% for *PNPLA3 148* locus G allele and 6.69% for *TM6SF2 167* locus T allele*.* The *PNPLA3 I148M* variant was significantly associated with the risk of NAFLD in an additive model (CG, OR = 2.092, 95% CI: 1.551–2.820, *P* = 0.000; GG, OR = 4.566, 95% CI: 3.141–6.638, *P* = 0.000, respectively). And, our data suggested a strong link between the *TM6SF2 E167K* variant and the risk of NAFLD in a dominant model (CT + TT, OR = 2.327, 95% CI: 1.542–3.513, *P* = 0.000). In addition, the increasing of the number of risk alleles were associated with the risk of NAFLD (1 risk allele, OR = 1.687, *P* = 0.001; 2 risk alleles, OR = 4.326, *P* = 0.000; 3 risk alleles, OR = 6.018, *P* = 0.027, respectively).

**Conclusions:**

Combining the *I148M* and *E167K* variants in a manner of an additive effect could improve risk prediction for NAFLD in a Qingdao Han Population cohort.

**Trial registration:**

Chinese Clinical Trial Register.gov: ChiCTR1800015426.

## Background

Nonalcoholic fatty liver disease (NAFLD) is identified as a metabolic stress injury of the liver, as well as a burgeoning public health issue/economic burden with a high global prevalence of up to ~ 25% [1, 2]. The incidence of NAFLD corresponds to the escalation of obesity, type 2 diabetes mellitus and metabolic syndrome [3]. Various evidence has indicated the heritable contribution to the prevalence, development and progression of NAFLD. The last few decades witness the substantial progress in the genetic susceptibility of NAFLD, providing exciting insight in refining the management and treatment of NAFLD patients. A recent prospective twin study performed by Loomba R et al. concluded that hepatic steatosis and hepatic fibrosis have approximately 52% heritability and 50% heritability in multivariable-adjusted models (adjust for sex, age and ethnicity), respectively [4]. However, we cannot clarify the molecular genetic mechanisms of NAFLD by only gene polymorphism [5].

Notably, two ‘star gene’ variants, *PNPLA3 I148M* variant and *TM6SF2 E167K* variant, are recognized as the major genetic modifiers of NAFLD [6–10]. More recently, the EASL-EASD-EASO clinical practice guidelines for the management of NAFLD recommended that carriers of the two ‘star gene’ variants show a higher liver fat content and increased risk of nonalcoholic steatohepatitis [11]. Interestingly, recent studies have demonstrated that *PNPLA3 I148M* and *TM6SF2 E167K* variants may have an additive effect in the regulation of lipid metabolism [8, 12–14]. However, no study has focused on the aforementioned association in Qingdao Han Population, China, an international city with more than ~ 9.2 million people after the 18th Meeting of the Council of Heads of Member States of the Shanghai Cooperation Organization. The present study sought to investigate the potential additive effect of the two variants on the risk of NAFLD in Qingdao Han Population, China.

## Methods

### Subjects and methods

The present study was conducted in accordance with the principles of the World Medical Association Declaration of Helsinki. All the subjects have signed written informed consent according to the study protocol approved by the Ethics Committee of Qingdao Municipal Hospital before participation (Approval NO.2017–20).

We recruited a total of 512 unrelated Qingdao Han NAFLD patients determined by liver ultrasonography (a fairly reliable and accurate noninvasive method with sensitivity of 84.8% and specificity of 93.6% [15]) and 451 healthy controls matched for age and sex in the present study. The healthy controls were performed liver ultrasonography to rule out NAFLD. All the subjects were of Qingdao Han ethnicity, China and included from the health examination center and the Department of Gastroenterology of Qingdao Municipal Hospital. The diagnosis of NAFLD was determined by a same experienced operator in accordance with the guidelines for management of NAFLD of the Chinese Liver Disease Association in 2010 [16]. Other etiologies contributing to fatty liver disease, including alcohol consumption (≥ 20 g/day for females and ≥ 30 g/day for males), hepatitis B infection, hepatitis C infection (genotype 3), autoimmune hepatitis, Wilson’s disease and drug-induced liver injury et al. were excluded [3].

Blood samples were collected after 12-h fast for serological assays and genetic analysis. Serum concentration of albumin (ALB), fasting plasma glucose (FPG), alanine aminotransferase (ALT), aspartate aminotransferase (AST), γ-glutamyltransferase (GGT), alkaline phosphatase (ALP), triglyceride (TG), total cholesterol (TC), high-density lipoprotein (HDL) and low-density lipoprotein (LDL), uric acid (UA) were obtained using standard methods in the Central Laboratory of Qingdao Municipal Hospital.

### Genotyping

Genomic DNA Purification Kit (BioTeke, Biotechnology, Beijing, China) was used to extract genomic DNA according to the manufacturer’s protocol from the noncoagulated blood samples. The genotyping of *PNPLA3 I148M* variant and *TM6SF2 E167K* variant was performed by polymerase chain reaction (PCR) with the following primers. Primers for *PNPLA3 I148M* and *TM6SF2 E167K* were: 5′- AACTTCTCTCTCCTTTGCTTTCACA -3′ (forward), 5′- GGAGGGATAAGGCCACTGTAGA -3′(reverse); 5′- TGTCTCAGAACAAACAAACAAACAGA -3′ (forward), 5′- GTAGGGGATGGTGAGGAAGAAG -3′ (reverse). The PCR amplification profile was conducted as follows: 95 °C for 10 min, 40 cycles before denaturation at 94 °C for 1 min, annealing at 58 °C for 1 min and elongation 30 s at 70 °C. The *PNPLA3* and *TM6SF2* genotypes were detected by ABI 3730XL (Foster City, CA, USA) and calculated by Gene Mapper 4.1 software.

### Statistical analysis

SPSS 20.0 statistical software (SPSS Inc., Chicago, IL, USA) was performed for statistical analysis. The baseline characteristics between healthy controls and NAFLD patients were showed as mean ± standard deviation, and the differences were examined using student’s *t* test or paired samples *t* test. The Hardy-Weinberg equilibrium was measured using the *χ*^*2*^ test. Genotype and allele frequencies between healthy controls and NAFLD patients were analyzed by chi-square test. The DNA distributions between the two groups were determined by *χ*^*2*^ test and Fisher’s exact test where appropriate. The association between the *PNPLA3 I148M* and/or *TM6SF2 E167K* variants and NAFLD was evaluated by the odds ratio (OR) with 95% confidence interval (CI) and performed by logistic regression analysis. In addition, an additive model (by coding the genotypes 0, 1 and 2 for CC, CG and GG, respectively) for *PLPLA3* gene and a dominant model (by coding the genotypes 0 and 1 for CC and CT + TT, respectively) for *TM6SF2* gene were assumed to assessed the potential additive effect of the two variants. Statistical significance was defined as *P*-value less than 0.05.

## Results

### Demographic and clinical characteristics

Demographic and clinical characteristics of healthy controls and NAFLD patients were presented in Table 1. When compared to the healthy controls, NAFLD patients had higher BMI, serum levels of FPG, ALT, AST, GGT, ALP, TG, TC, LDL, UA and decreased ALB, HDL. All the difference reached significance (all *P* < 0.05).

**Table 1:** Demographic and clinical characteristics of healthy controls and nonalcoholic fatty liver disease patients

N	Healthy controls	NAFLD patients	*t*	*P* value
	451	512		
BMI(kg/m^^2^)	22.48 ± 3.12	26.71 ± 2.81	−21.967	0.000
ALB (g/L)	46.78 ± 2.31	46.28 ± 2.84	2.980	0.003
FPG (mmol/L)	4.69 ± 2.67	4.99 ± 1.28	−2.219	0.027
ALT (U/L)	20.11 ± 15.07	36.98 ± 23.78	−13.300	0.000
AST (U/L)	21.06 ± 8.91	26.34 ± 11.33	−8.081	0.000
GGT (U/L)	24.20 ± 23.73	46.79 ± 35.02	−11.833	0.000
ALP (U/L)	62.19 ± 15.43	77.00 ± 26.29	−10.808	0.000
TG (mmol/L)	1.20 ± 1.04	2.16 ± 1.64	−11.021	0.000
TC (mmol/L)	5.18 ± 0.88	5.49 ± 1.12	−4.690	0.000
HDL (mmol/L)	1.42 ± 0.29	1.25 ± 0.24	9.529	0.000
LDL (mmol/L)	2.93 ± 0.69	3.70 ± 1.26	−11.423	0.000
UA (mmol/L)	309.16 ± 74.02	401.13 ± 88.05	−17.545	0.000

*Abbreviations*: *NAFLD* nonalcoholic fatty liver disease, *BMI* body mass index, *ALB* albumin, *FPG* fasting plasma glucose, *ALT* alanine aminotransferase, *AST* aspartate aminotransferase, *GGT* γ-glutamyltransferase, *ALP* alkaline phosphatase, *TG* Triglyceride, *TC* total cholesterol, *HDL* high-density lipoprotein, *LDL* low-density lipoprotein, *UA* uric acid

### Associations between *PNPLA3 I148M* and *TM6SF2 E167K* variants and NAFLD

The genotypic distribution of *PNPLA3 I148M* and *TM6SF2 E167K* were both in Hardy-Weinberg equilibrium in both two groups (*P*_control_ = 0.243, 0.773; *P*_NAFLD_ = 0.113, 0.313). The minor allele frequencies of the two single-nucleotide polymorphisms (SNPs) in the whole subjects were 45.48% for *PNPLA3 I148M* and 6.69% for *TM6SF2 E167K*, respectively*.* As listed in Table 2, both the differences in the genotypes and allele frequencies of the *PNPLA3 I148M* and *TM6SF2 E167K* reached significance (all *P* < 0.05). Moreover, Table 3 showed that the *PNPLA3 I148M* variant was significantly associated with the risk of NAFLD in an additive model (CG, OR = 2.092, 95% CI: 1.551–2.820, *P* = 0.000; GG, OR = 4.566, 95% CI: 3.141–6.638, *P* = 0.000, respectively). Interestingly, it suggested a strong link between the *TM6SF2 E167K* variant and the risk of NAFLD in a dominant model (CT + TT, OR = 2.327, 95% CI: 1.542–3.513, *P* = 0.000).

**Table 2:** Distribution of genotypes and allele frequencies of *I148M* and *E167K* variants in the whole subjects

	Controls *n* (%)	NAFLD *n* (%)	*χ* ^*2*^	*P* value
*PNPLA3 I148M*				
CC	196 (43.46)	114 (22.27)		
CG	194 (43.02)	236 (46.09)		
GG	61 (13.52)	162 (31.64)	67.946	0.000
Allele C	586 (64.97)	464 (45.31)		
Allele G	316 (35.03)	560 (54.69)	74.711	0.000
*TM6SF2 E167K*				
CC	415 (92.02)	426 (83.20)		
CT	35 (7.76)	80 (15.63)		
TT	1 (0.22)	6 (1.17)	17.530	0.000
Allele C	865 (95.90)	932 (91.02)		
Allele T	37 (4.10)	92 (8.98)	18.293	0.000

**Table 3:** Associations between *I148M* and *E167K* variants and nonalcoholic fatty liver disease

	OR (95% CI)	*P* value
*PNPLA3 I148M*		
CC	1	
CG	2.092 (1.551–2.820)	0.000
GG	4.566 (3.141–6.638)	0.000
*TM6SF2 E167K*		
CC	1	
CT + TT	2.327 (1.542–3.513)	0.000

Furthermore, Table 4 demonstrated the association between the two variants and clinical features. Subjects with the G allele of *PNPLA3 I148M* had higher BMI, serum levels of ALT, AST, GGT, ALP, TG, TC, LDL and UA (all *P* < 0.05). The serum level of HDL did not reached significance (*P* = 0.967). However, only serum concentrations of some lipid profiles (TG and TC; both *P* < 0.05) showed inversely strong links between carriers of the T allele of *TM6SF2 E167K* and the noncarriers. The T allele of *TM6SF2 E167K* was associated with lower levels of serum TG and TC (*P* = 0.003, *P* = 0.006).

**Table 4:** Association between *I148M* and *E167K* variants and clinical features in the whole subjects

	*PNPLA3 I148M*				*TM6SF2 E167K*			
	Noncarriers	Carriers	*t*	*P* value	Noncarriers	Carriers	*t*	*P* value
N	310	653			841	122		
BMI(kg/m^2)	24.17 ± 3.41	25.00 ± 3.70	−3.323	0.000	24.73 ± 3.64	24.75 ± 3.55	−0.053	0.958
ALB(g/L)	46.59 ± 2.22	46.47 ± 2.78	0.629	0.529	46.47 ± 2.38	46.82 ± 3.86	−0.997	0.321
FPG (mmol/L)	4.75 ± 1.21	4.90 ± 2.34	−1.064	0.288	4.87 ± 2.16	4.75 ± 1.02	−0.556	0.578
ALT (U/L)	23.68 ± 18.04	31.64 ± 23.02	−5.837	0.000	28.91 ± 22.31	30.25 ± 18.44	−0.634	0.527
AST (U/L)	21.86 ± 7.06	24.82 ± 11.81	−4.851	0.000	23.73 ± 10.57	24.83 ± 10.77	−1.070	0.285
GGT (U/L)	30.83 ± 25.99	38.77 ± 34.60	−3.960	0.000	36.68 ± 33.02	32.98 ± 26.55	1.185	0.236
ALP (U/L)	64.86 ± 15.67	72.53 ± 25.52	−5.731	0.000	69.72 ± 22.87	72.40 ± 24.54	−1.195	0.232
TG (mmol/L)	1.27 ± 0.81	1.92 ± 1.66	−8.124	0.000	1.75 ± 1.52	1.43 ± 0.99	3.057	0.003
TC (mmol/L)	5.25 ± 0.99	5.39 ± 1.04	−2.000	0.046	5.38 ± 1.05	5.15 ± 0.80	2.788	0.006
HDL (mmol/L)	1.33 ± 0.25	1.33 ± 0.28	0.042	0.967	1.32 ± 0.27	1.38 ± 0.33	−1.863	0.065
LDL (mmol/L)	3.03 ± 0.76	3.48 ± 1.20	−6.723	0.000	3.35 ± 1.12	3.23 ± 0.91	1.125	0.261
UA (mmol/L)	342.72 ± 94.88	365.84 ± 92.41	−3.581	0.000	359.04 ± 95.72	354.13 ± 79.58	0.619	0.536

*Abbreviations*: *BMI* body mass index, *ALB* albumin, *FPG* fasting plasma glucose, *ALT* alanine aminotransferase, *AST* aspartate aminotransferase, *GGT* γ-glutamyltransferase, *ALP* alkaline phosphatase, *TG* triglyceride, *TC* total cholesterol, *HDL* high-density lipoprotein, *LDL* low-density lipoprotein, *UA* uric acid

### Additive effects of *PNPLA3 I148M* and *TM6SF2 E167K* variants on the risk of NAFLD

**Fig. 1:**
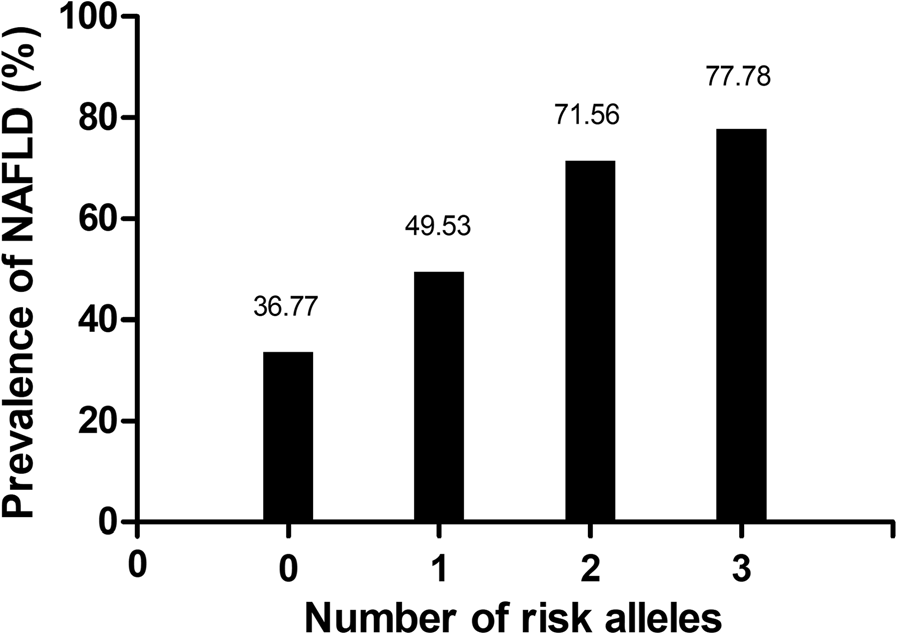
Prevalence of NAFLD according to the number of risk alleles Prevalence of NAFLD according to the number of risk alleles is shown. We counted risk alleles at the two gene locus (for *PNPLA3* 148 locus, by coding the genotypes 0, 1 and 2 for CC, CG and GG; for *TM6SF2* 167 locus, by coding the genotypes 0 and 1 for CC and CT + TT, respectively). The prevalence of NAFLD increased following with the increase of the number of risk allele.

Moreover, the potential additive effect of the two variants on the risk of NAFLD was conducted in the study subjects. We counted risk alleles at the two gene locus (for *PNPLA3* 148 locus, by coding the genotypes 0, 1 and 2 for CC, CG and GG; for *TM6SF2* 167 locus, by coding the genotypes 0 and 1 for CC and CT + TT, respectively). The group with 0 risk allele was regarded as the control group. Excitingly, the prevalence of NAFLD increased following with the increase of the number of risk alleles (36.77, 49.53, 71.56 and 77.78% of subjects with 0, 1, 2 and 3 risk alleles, respectively, Fig. 1). In addition, the increasing of the number of risk alleles showed a strong link with the risk of NAFLD (1 risk allele, OR = 1.687, 95% CI: 1.226–2.321, *P* = 0.001; 2 risk alleles, OR = 4.326, 95% CI: 3.100–6.037, *P* = 0.000; 3 risk alleles, OR = 6.018, 95% CI: 1.229–29.459, *P* = 0.027, respectively, Table 5).

**Table 5:** OR (95% CI) for nonalcoholic fatty liver disease in subjects with different number of risk alleles

Risk alleles (n)	OR (95%CI)	*P* value
0	1	
1	1.687 (1.226–2.321)	0.001
2	4.326 (3.100–6.037)	0.000
3	6.018 (1.229–29.459)	0.027

## Discussion

Our study aimed to assess the potential additive effect of the *PNPLA3 I148M* and *TM6SF2 E167K* variants on the risk of NAFLD in Qingdao Han Population, China. We confirmed the significant association between both the *PNPLA3 I148M* and *TM6SF2 E167K* variants and the risk of NAFLD in a cohort consisting of a total of 512 NAFLD patients and 451 age and sex matched healthy controls. Moreover, combining the *I148M* and *E167K* variants could improve risk prediction for NAFLD in a Qingdao Han Population.

NAFLD ranked as the most concerned chronic liver disease in the coming decades worldwide, and, nonalcoholic steatohepatitis is recognized as the second leading indication for liver transplantation in the USA [17–19]. Notably, there is a genetic susceptibility for NAFLD [1, 3, 11]. Mahdessian H et al. firstly reported that *TM6SF2* inhibition induced significant reduction of the expression of *PNPLA3* gene in both Huh7 and HepG2 cells, which, to some extent, indicating a joint/additive effect between *PNPLA3* gene and *TM6SF2* gene in determining lipid metabolism [20]. More recently, a multiethnic study [12] of obese children and adolescents (including Caucasians, African Americans, and Hispanics) summarized that there is a joint effect among *PNPLA3 I148M*, *TM6SF2 E167K*, and *GCKR rs1260326* single nucleotide polymorphisms in regulating intrahepatic fat accumulation. Similarly, Wang and colleagues [13] demonstrated the additive effect of *PNPLA3 I148M* and *TM6SF2 E167K* variants on NAFLD in a Chinese cohort. In the present study, we observed that *PNPLA3 I148M* variant and *TM6SF2 E167K* variant were both major and independent genetic determinants of NAFLD in a Qingdao Han Population, consistent with previous studies [21, 22]. More importantly, subjects in our study with increasing number of risk alleles (*PNPLA3 148* locus G allele and *TM6SF2 167* locus T allele) showed an additive effect of the two variants, compared to individuals with 0 risk allele (1 risk allele, OR = 1.687, *P* = 0.001; 2 risk alleles, OR = 4.326, *P* = 0.000; 3 risk alleles, OR = 6.018, *P* = 0.027, respectively). Furthermore, our pilot study [23] performed a Bayesian analysis and evaluated the additive effect of the two variants on hepatocyte lipid metabolism as well as the underlying mechanism in vitro. Our date suggested that the *PNPLA3 I148M* and *TM6SF2 E167K* variants may increase hepatic lipid content by upregulating the expression of sterol regulatory element-binding transcription factor 1c and fatty acid synthase. However, to the best of our knowledge, all other previous studies have not reported the underlying molecular mechanism of this potential additive effect. These encouraging findings provide the potential perspective in new NAFLD classification and the probability of offering a new important tool for risk prediction of NAFLD in the coming years.

We additionally investigated the relations between *PNPLA3 I148M* and *TM6SF2 E167K* variants and serum concentrations of lipid profiles. Several studies showed inconsistent metabolic associations of NAFLD risk alleles. For instance, some studies demonstrated a strong link between *TM6SF2 E167K* variant and lower plasma levels of TC, TG and LDL [8, 24–26], but the differences in several other studies did not reach the significance [9, 27, 28]. Similarly, the association between the *PNPLA3 I148M* variant and plasma concentrations of lipid profiles was also inconsistent. [6, 29–31]. Our data demonstrated that subjects with the G allele of *PNPLA3 I148M* had higher serum levels of TG, TC and LDL (all *P* < 0.05). And, the serum concentrations of some lipid profiles (TG and TC; both *P* < 0.05) showed inversely strong links between carriers of the T allele of *TM6SF2 E167K* and the noncarriers. The T allele of *TM6SF2 E167K* was associated with lower concentrations of serum levels of TG and TC (*P* = 0.003, *P* = 0.006). These different findings may inasmuch as the population ethnicity, diagnostic method of NAFLD and demographic and clinical characteristics. Therefore, the relationship between the two ‘star gene’ variants and clinical features of NAFLD population needs further investigation.

This study has some limitations. First one is the perform of ultrasonography to determine NAFLD, instead of magnetic resonance imaging and/or liver biopsy. Notably, liver ultrasonography cannot provide reliable quantitative data [32]. In addition, we conducted the present study in a single center and the findings may have limited application value in other different populations. Furthermore, the size of subjects in the study was not sufficiently large to comprehensively explore the associations.

## Conclusions

In conclusion, we confirmed the significant association between both the *PNPLA3 I148M* and *TM6SF2 E167K* variants and the risk of NAFLD in a Qingdao Han Population cohort. More importantly, combining the *I148M* and *E167K* variants could improve risk prediction for NAFLD in a manner of an additive effect. This study may provide the potential perspective in new NAFLD classification and the identification of high risk NAFLD populations in the future.

## Abbreviations

95% CI: 95% confidence interval
ALB: Albumin
ALP: Alkaline phosphatase
ALT: Alanine aminotransferase
AST: Aspartate aminotransferase
BMI: Body mass index
FPG: Fasting plasma glucose
GGT: γ-glutamyltransferase
HDL: High-density lipoprotein
LDL: Low-density lipoprotein
NAFLD: Nonalcoholic fatty liver disease
OR: Odds ratio
PCR: Polymerase chain reaction
TC: Total cholesterol
TG: Triglyceride
UA: Uric acid
